# Biopolymer Meets Nanoclay: Rational Fabrication of Superb Adsorption Beads from Green Precursors for Efficient Capture of Pb(II) and Dyes

**DOI:** 10.3390/nano14090766

**Published:** 2024-04-26

**Authors:** Jie Qi, Xue Wang, Huan Zhang, Xiangyu Liu, Wenbo Wang, Qingdong He, Fang Guo

**Affiliations:** College of Chemistry and Chemical Engineering, Inner Mongolia University, Hohhot 010021, China; jieino@163.com (J.Q.); wx15847261836@163.com (X.W.); hzhang1989@163.com (H.Z.); lxy13257244737@163.com (X.L.); wangwenbo@imu.edu.cn (W.W.); 18847744604@163.com (Q.H.)

**Keywords:** alginate, rectorite, bead, adsorption, wastewater

## Abstract

Renewable, green, and safe natural biopolymer-derived materials are highly desired for the purification of pollutants, but significantly improving their performance without the introduction of additional harmful chemicals remains a huge challenge. Based on the concept of “structure optimization design”, environment-friendly composite beads (named SA/PASP/RE) with excellent adsorption performance and recyclability were rationally constructed through a green ionic crosslinking route, using the completely green biopolymer sodium alginate (SA), sodium salt of polyaspartic acid (PASP), and the natural nanoclay rectorite (RE) as starting materials. The nano-layered RE was embedded in the polymer matrix to prevent the polymer chain from becoming over-entangled so that more adsorption sites inside the polymer network were exposed, which effectively improved the mass transfer efficiency of the adsorbent and the removal rate of contaminants. The composite beads embedded with 0.6% RE showed high adsorption capacities of 211.78, 197.13, and 195.69 mg/g for Pb(II) and 643.00, 577.80, and 567.10 mg/g for methylene blue (MB) in Yellow River water, Yangtze River water, and tap water, respectively. And the beads embedded with 43% RE could efficiently adsorb Pb(II) and MB with high capacities of 187.78 mg/g and 586.46 mg/g, respectively. This study provides a new route to design and develop a green, cost-effective, and efficient adsorbent for the decontamination of wastewater.

## 1. Introduction

With the rapid development of battery, electroplating, metal metallurgy, textile, papermaking, plastic, coating, and other industries, it is inevitable that a large amount of wastewater containing heavy metals and dyes will be generated and discharged, causing serious pollution to rivers, groundwater, soil, and other environments, which has become one of the environmental safety issues of global concern [1,2]. Water pollution is becoming more and more serious, and it will pose a major threat to the safety of human beings and other living beings on Earth [3,4]. It has been proven to be the culprit of many serious human diseases, such as cancer, gene mutations, malformations, superbugs, and so on. Therefore, decontaminating wastewater is required by developing more materials or technologies to minimize its harm to human health.

In order to meet the needs of purifying increasingly diverse pollutants, such as dyes or heavy metals, various chemical and physical methods have been developed and adopted, such as chemical precipitation [5], catalytic degradation [6,7], filtration [8], electrochemistry [9], and adsorption [10,11]. These methods have played an important role in the removal of pollutants with their respective advantages, but people have not stopped exploring “all-around soldiers” that can be used for the efficient removal of multiple pollutants. Many commonly recognized weaknesses in existing methods limit their use as “all-around soldiers” for the efficient removal of multiple pollutants. For example, the chemical precipitation method is powerless to remove low concentrations of pollutants and a large number of chemical reagents have to be used, which may cause the risk of secondary pollution. The catalytic degradation method is applicable to organic pollutants but not to pollutants that cannot be degraded, such as heavy metal ions. The costs of electrochemical and ultrafiltration methods are relatively higher. By contrast, the adsorption process is simple and low cost. It is not only applicable for organic pollutants but also for the efficient removal of non-degradable pollutants such as heavy metal ions [12,13], even at very high or low concentrations [14,15]. More importantly, all pollutants can be completely removed from wastewater by a one-step adsorption process without secondary pollution, so the adsorption method stands out among many methods.

The advantages and application potential of the adsorption method in pollutant removal have stimulated the rapid development of adsorbents. So far, various adsorption materials with different functions have been developed, such as polymer composites [16,17], porous silicate [18], graphene oxide [19], sisal fiber felt [20], aerogels [21], clay@carbon composite [22], etc. Many synthetic materials show very high adsorption capacities due to their unique pore structure and rich functional groups. However, as “green and sustainable development” has become the theme of future development, the environmental friendliness of the materials themselves has been paid more and more attention. However, at present, most adsorption materials are developed with the adsorption capacity as the goal, and the environmental friendliness of the materials themselves has not been fully considered; thus, new attention has been placed on the development of new adsorption materials that are both high performance and environmentally friendly [23]. Through the analysis of the existing literature, it can be concluded that, among many types of materials, the adsorption materials prepared from naturally available raw materials (such as natural polymers and clay minerals) are the most promising because they are expected to obtain relatively better performance with the premise of ensuring environmental friendliness. However, in the actual process, how to modify or optimize the design of natural materials to obtain high-performance, green adsorption materials is still a huge challenge.

Natural biopolymers have attracted more and more attention for the preparation of environmentally friendly adsorbents due to their advantages of renewability, environmental friendliness, ease of modification, functional regulation, and high biodegradability [24,25]. Sodium alginate (SA) is a renewable, biodegradable, non-toxic, and biocompatible natural polysaccharide extracted from brown algae [26]. The abundant hydroxyl and carboxyl groups on the macromolecular chains of SA enable it to be crosslinked by various crosslinkers, blended with other components, or modified by a grafting process to form new materials capable of capturing metal ions or organic molecules by complexing or electrostatic interaction [27,28]. Therefore, SA has been recognized as the first choice for economical, efficient, and environmentally friendly composite adsorbent materials. Polyaspartic acid is an amino acid polymer composed of L-aspartic acid as a monomer. With multiple amide bonds formed by the condensation of amino and carboxyl groups on the main chain and multiple carboxyl groups on the side chain, PASP is a unique and environmentally friendly water-soluble polymer material with good biocompatibility and biodegradability [29]. In industry, it is often used as a green water reducing agent [30], drug delivery carrier [31], green corrosion inhibitor [32], water retaining agent [33], adsorbent [34], etc. Because the PASP molecular chain contains a large number of functional groups that can be complexed with metal ions, PASP is a potential raw material for the preparation of metal ion adsorbents. With the industrialization of L-aspartic acid production by biotechnology in recent years, it is possible to use PASP to produce high-performance, environmentally friendly adsorption materials.

The research on natural polymer adsorbents has rapidly developed in recent years due to their excellent environmental friendliness, but pure organic adsorbents are still faced with the problem of low removal rates, and it is difficult to completely remove pollutants from water due to poor mass-transfer efficiency [35]. The combination of natural polymers with other nanomaterials is an effective method to improve their adsorption and removal ability [36]. Natural clay minerals are a class of porous silicate less than 2 microns in size that are composed of safe elements with the highest abundance on Earth, such as silicon, oxygen, aluminum, magnesium, iron, and potassium. They have unique nanostructural units and surface properties, such as a permanent structural charge, controllable interlayer domain, special pore structure, and active surface groups [37,38]. Clay minerals are widely distributed around the world with abundant reserves and natural origins, so they have the advantages of excellent performance, low price, and environmental friendliness, and they show broad application prospects in environmental treatments such as wastewater, waste gas, and soil remediation [39,40]. Rectorite (RE) is an interlayered clay mineral arranged with the montmorillonite layer and the mica layer in a 1:1 arrangement. With large layer spacing and strong ion exchange ability, RE shows excellent adsorption performance for dyes or metal ions [41,42]. At the same time, RE has good environmental friendliness, with its use causing no secondary harm, and is an ideal raw material for the preparation of environmentally friendly adsorption materials; thus, it has attracted more and more attention in recent years.

To this end, in view of the defects of synthetic adsorbents, such as environmental unfriendliness, and the low adsorption capacities and poor adsorption and removal abilities of traditional natural adsorbents, this study adopts the dual design of molecular structure and nanocomposite structure, using natural sodium alginate (SA), polymeric aspartic acid (PASP), and natural rectorite (RE) as raw materials. The SA/PASP/RE composite adsorption beads with low cost, environmental protection, easy separation, and high performance, composed of all-natural components, were prepared by a simple green crosslinking method and were used for the adsorption and removal of dyes and heavy metal pollutants in water. The structure of the adsorbent was characterized systematically, the adsorption properties of the adsorbent were evaluated, and the adsorption mechanism was discussed.

## 2. Experimental

### 2.1. Materials and Reagents

SA [molecular formula: (C_6_H_7_O_6_Na)n; CAS number: 9005-38-3; MDL: MFCD0081310] and calcium chloride (CaCl_2_) (A.R. grade) were purchased from Inno Chem Reagent Co., Ltd., Beijing, China. SA was of analytical reagent grade and had a sodium content of 7.31 wt.%, molecular weight (MW) of 65,340 Da, and distribution index PD value of 3.047. Sodium salt of polyaspartic acid (PASP) (40% aqueous solution) was purchased from Shandong Yousuo Chemical Technology Co., Ltd., Heze, China. Rectorite (RE) was kindly provided by Hubei Mingliu Rectorite Co. Ltd. (Zhongxiang, China). Methylene blue (MB) and Pb(NO_3_)_2_ were purchased from Adamas-beta Co., Ltd. (Shanghai, China). The solvent used for preparing the beads was deionized water. The Yangtze River water sample was sourced from Jingzhou, China. The Yellow River water sample was sourced from Ordos City, China. The seawater was sourced from the Bohai Sea (Tianjin, China).

### 2.2. Preparation of SA/PASP/RE Composite Beads

**Scheme 1:**
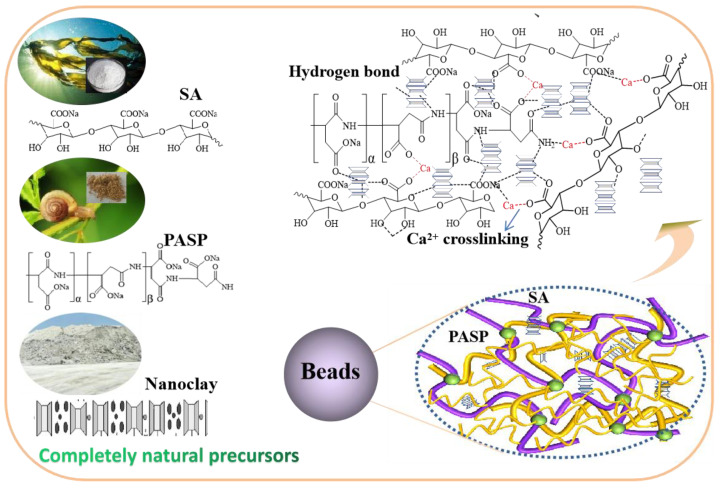
The scheme illustrating the natural raw materials and network structure.

SA powder (1.0 g) was completely dissolved in 50 mL of deionized water under continuous mechanical stirring to obtain a homogeneous aqueous solution of SA. Then, PASP and RE (0.025 g, 0.05 g, 0.075 g, and 0.1 g, respectively) were added to the aqueous solution of SA. The resulting mixture was dropped into the aqueous solution of CaCl_2_ with a syringe to form Ca^2+^-crosslinked beads. The beads were filtered out with a stainless steel sieve, immersed in 70% ethanol for dehydration, and then dried to obtain the composite adsorption beads composed of natural components. The obtained adsorption beads were labeled as SA/PASPx/REy [x is PASP content (x%), and y is the content of RE (y%)]. By examining the adsorption capacity of the beads for MB and Pb(II) under different conditions, the optimal content of RE in the beads was found to be 0.6%, and the optimal sample was labeled as SA/PAM/RE0.6. The apparent morphology of the composite beads is depicted in Figure S1 (Supplementary Material), and the preparation process of the beads and the Ca^2+^-crosslinked network structure are depicted in Scheme 1.

### 2.3. Adsorption Experiments

The adsorption performance of the composite beads was evaluated through batch adsorption experiments. The beads were mixed with 20 mL of aqueous solution containing MB or Pb(II), and then the mixture was placed in a constant temperature shaker (THZ-98A, Yiheng, Shanghai, China) and fully shaken at 120 rpm for 10 h at 30 °C. In the optimization experiment of the preparation conditions, the initial concentrations of MB and Pb(II) solutions were 400 mg/L and 300 mg/L, respectively. After adsorption, the concentration of dye or Pb(II) in the solution was measured with a UV–Vis spectrophotometer (UV1900i, Shimadzu) and atomic absorption spectrometer (Analytic Jena, Jena, Germany), respectively. The saturated adsorption capacity (*q*_e_) of the beads for cationic dye and Pb(II) was calculated using Equation (1).

For adsorption kinetic testing, 0.01 g of adsorption beads was thoroughly mixed with 20 mL of MB solution (concentration: 200 mg/L) or Pb(II) solution (concentration: 400 mg/L) at 30 °C. The adsorption capacity of the adsorbent was measured at different contact times (5, 15, 30, 60, 120, 180, 240, 360, 480, and 600 min, respectively), and the dependence curve of adsorption capacity against time was plotted. When conducting adsorption kinetic tests, the adsorption capacity (*q*_t_) of Pb(II) and cationic dye at time interval t was calculated using Equation (2). For adsorption isotherm testing, 0.01 g of adsorbent beads was fully exposed to 20 mL of MB or Pb(II) solution with different initial concentrations (100, 200, 300, 400, 500, 600, and 700 mg/L, respectively) at 30 °C, and the adsorption capacity was tested under different initial concentration conditions. The dependence curve of adsorption capacity against concentration was plotted. The removal efficiency for MB or Pb(II) from aqueous solution by the adsorption beads was examined under different doses of adsorption beads, and the removal efficiency was calculated using Equation (3).

*q*_e_ = [(*C*_0_ − *C*_e_) × *V*]/*m* (1)

*q*_t_ = [(*C*_0_ − *C*_t_) × *V*]/*m* (2)

Removal efficiency (%) = [(*C*_0_ − *C*_e_)/*C*_0_] × 100% (3)

In these equations, *C*_0_ is the original concentration of MB or Pb(II) solution (unit: mg/L). *C*_e_ is the concentration of MB or Pb(II) solution at the saturation adsorption state (mg/L). *V* is the volume of solution (L). *m* is the mass of adsorbent used (g).

### 2.4. Adsorption of MB and Pb(II) in Different Natural Waters

Aqueous solutions of MB or Pb(II) were prepared using tap water, Yellow River water, and Yangtze River water as the solvents, and the adsorption effects of the adsorption beads toward MB and Pb(II) in different natural water samples were evaluated. In the adsorption experiment of Pb(II), due to the pH value of natural water being about 6, some Pb(II) would precipitate out in the form of Pb(OH)_2_. Therefore, the pH value of the Pb(II) solution should be controlled at around 4 to ensure the accuracy of the evaluation results. The initial concentration of the MB solution was 400 mg/L, which was prepared by directly dissolving dye in natural water samples for the adsorption experiments (adsorption conditions: adsorbent dosage, 0.5 g/L; solution volume, 20 mL; adsorption temperature, 30 °C). The adsorption performance of the beads for MB and Pb(II) in natural water was evaluated according to the procedure described above.

### 2.5. Desorption and Recycling Experiment

The MB dye adsorbed on the adsorption beads was desorbed, and the beads were reused to study their recycling and regeneration performance. Firstly, the adsorption beads (0.01 g) were mixed with 20 mL of MB solution (concentration: 400 mg/L) in a plastic centrifuge tube, which was then placed in a constant temperature shaker and continuously shaken for 10 h at 120 rpm and 30 °C. After washing the weakly adsorbed MB on the surface of the beads with distilled water, the beads were immersed in 20 mL of mixed solution of hydrochloric acid/ethanol (0.5 mol/L HCl + 80% ethanol), and the mixture was shaken for 12 h in a constant temperature shaker to ensure the complete desorption of MB. The concentration of MB in the solution was determined using UV–Visible spectroscopy. The desorption amount of the MB dye was calculated. The desorption rate (%) was calculated using Equation (4). Finally, the regenerated beads were used again for the absorption of MB.

(4) Desorption rate (%)=(Desorption amount/Adsorption amount)×100%

### 2.6. Characterization

The samples were characterized using the instruments and methods described in the Supplementary Material.

## 3. Results and Discussion

### 3.1. Structure and Morphology of Beads

**Figure 1:**
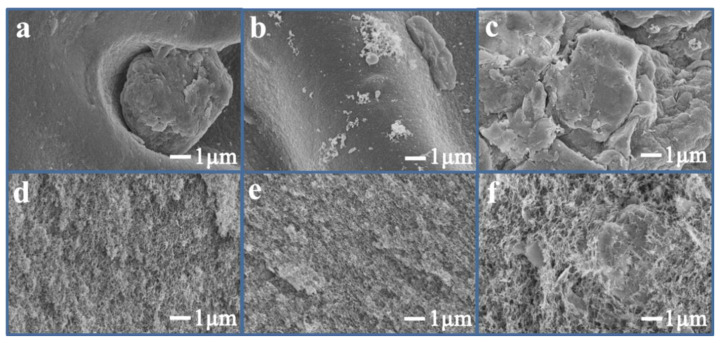
SEM images of the outer surface of (**a**) SA/PASP, (**b**) SA/PASP/RE0.6, and (**c**) SA/PASP/RE43; cross-sections of composite beads: (**d**) SA/PASP, (**e**) SA/PASP/RE0.6, and (**f**) SA/PASP/RE43.

**Figure 2:**
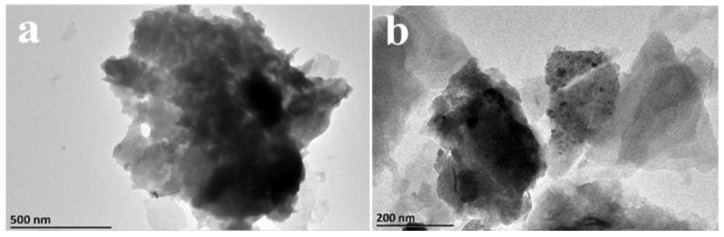
TEM images of the cross-sections of (**a**) SA/PASP/RE0.6 and (**b**) SA/PASP/RE43 beads.

The surface morphology, microstructure, and surface element distribution of the composite beads were studied by SEM (Figure 1), TEM (Figure 2), and EDS (Figure S2) analyses, respectively. With the increase in RE content, the surface roughness of the beads increased. Compared with the SA/PASP beads, the SA/PASP/RE43 composite beads exhibited a porous structure, and large amounts of loose network pores could be clearly found in the SEM image of the cross-section. The active hydroxyl group on RE may have formed a strong intermolecular hydrogen bonding interaction with the SA and PASP chains, and Ca^2+^ was crosslinked with the carboxyl and amino groups in the polymer chain through metal complexation, which increased the crosslinking density on the outer surface of the beads and formed an extremely smooth surface morphology. By contrast, a lower crosslinking density was more conducive to the formation of a loose and porous structure in the beads.

From the TEM image of SA/PASP/RE0.6 (Figure 2), no obvious aggregation of RE particles was observed, indicating that RE was uniformly dispersed in the polymer matrix at a low addition amount. In the TEM image and EDS diagram of SA/PASP/RE43 (Figure 2 and Figure S1 in Supplementary Material), a small amount of inorganic particles was observed, indicating that some RE particles existed in the polymer network in the form of physical filling when the addition amount of RE was high. The EDS and element distribution analysis results showed that Si, Al, O, and Mg elements were evenly distributed on the surface of the SA/PASP/RE0.6 and SA/PASP/RE43 beads, which confirmed the better compatibility of RE with the polymer matrix, and the organic-inorganic compound structure was formed [43].

**Figure 3:**
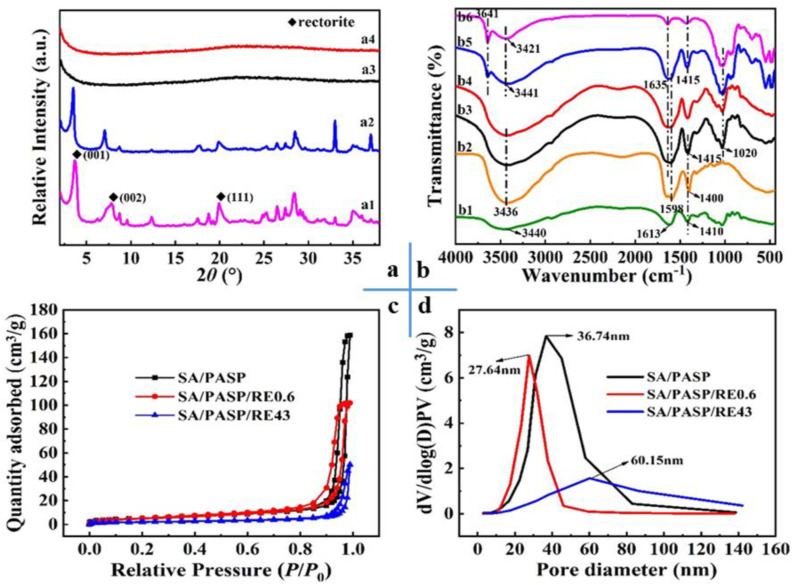
XRD patterns of (**a**): (a1) RE, (a2) SA/PASP/RE43, (a3) SA/PASP/RE0.6, (a4) SA/PASP; FT-IR spectra of (**b**): (b1) SA, (b2) PASP, (b3) SA/PASP, (b4) SA/PASP/RE0.6, (b5) SA/PASP/RE43, and (b6) RE; (**c**) N_2_ adsorption–desorption isotherms; and (**d**) pore size distribution curves of SA/PASP, SA/PASP/RE43, and SA/PASP/RE0.6.

Figure 3(a1–a4) shows the XRD patterns of RE, SA/PASP/RE43, SA/PASP/RE0.6, and SA/PASP samples. The characteristic diffraction peaks with 2θ values of 3.8°, 7.9°, and 19.8° appeared in the XRD pattern of RE, corresponding to the (001), (002) and (111) crystal planes of rectorite, respectively [44]. When a small amount of RE was introduced to form the SA/PASP/RE0.6 composite beads, the characteristic diffraction peak of RE was not observed in the XRD pattern due to the low content of RE and easy peeling during the composite process. However, when more RE was introduced, the characteristic diffraction peaks of RE appeared in the XRD pattern of SA/PASP/RE43, indicating that an excess amount of RE was present in the polymer matrix by physical filling and combined with the network through hydrogen bonding interaction.

The types and states of chemical groups on the surface of the composite adsorption beads were studied by FT-IR spectroscopy. From the FT-IR spectrum of SA, characteristic absorption peaks at 3440 cm^−1^ (-OH stretching vibration), 1613 cm^−1^ (asymmetric stretching vibration of -COO), and 1417 cm^−1^ (symmetric stretching vibration of -COO) could be observed (Figure 3(b1)) [45,46]. In the FT-IR spectrum of PASP (Figure 3(b2)), characteristic absorption peaks could be observed at 3436 cm^−1^ (stretching vibration peak of N-H bond on amide group), 1598 cm^−1^ (antisymmetric stretching vibration peak of carboxylate group), and 1400 cm^−1^ (symmetric stretching vibration peak of carboxylate group). The absorption peak of SA/PASP at 3428 cm^−1^ was the overlapping peaks of OH and -NH_2_ of SA and PASP [47,48]. From the FT-IR spectrum of RE (Figure 3(b6)), peaks could be observed at 3641 cm^−1^ (SiO-H and AlO-H stretching vibration peaks), 1020 cm^−1^ (in-plane Si-O-Si stretching vibration peaks), 3405 cm^−1^ (H-O-H stretching vibration of water molecules), and 1635 cm^−1^ (H-O-H stretching vibration peaks). The wide absorption peak was observed at the position of the flexural vibration peak of the bond [49,50]. After calcium ions were crosslinked, the -COO- on the molecular chains of SA and PASA complexed with calcium ions, which linked different molecular chains together to form an interconnected network structure. The absorption peak of SA/PASP at 1415 cm^−1^ was the superposition peak of the absorption peak of SA at 1417 cm^−1^ and the absorption peak of PASP at 1400 cm^−1^ (Figure 3(b3)). After RE was introduced to form the SA/PASP/RE0.6 and SA/PASP/RE43 composite adsorption beads, the O-H stretching vibration peak of SA/PASP shifted from 3441 cm^−1^ to 3421 cm^−1^ (Figure 3(b4–b5)). This indicated that the surface silanol group (Si-OH) of RE formed hydrogen bonds with the hydroxyl or carboxyl groups on the SA and PASP chains.

The N_2_ adsorption–desorption isotherms and pore size distributions of the SA/PASP and SA/PASP/RE composite adsorption beads are shown in Figure 3c,d. According to IUPAC classification, all samples showed typical IV isotherms with H3 loops (Figure 3c), indicating the presence of mesoporous structures in the samples [51]. The introduction of a small amount of RE narrowed the pore size distribution of the composite beads (Figure 3d). The maximum pore size distribution peak appeared at 27.64 nm, showing more obvious mesoporous characteristics. By optimizing the RE content, we found that the pore size of the adsorption beads increased from 36.74 nm (SA/PASP) to 60.15 nm (SA/PASP/RE43). The introduction of a small amount of RE greatly increased the amount of pores, which was more conducive to the adsorption of dyes.

### 3.2. Adsorption Properties

#### 3.2.1. Effect of RE Content on Adsorption Capacity

**Figure 4:**
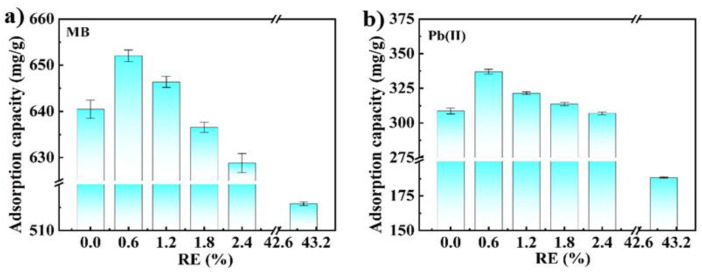
The effect of RE content on the adsorption capacity of the beads toward (**a**) MB and (**b**) Pb(II).

The all-natural composite adsorption beads with different RE contents (0 g, 0.025 g, 0.05 g, 0.075 g, 3 g) were prepared by combining natural RE clay with SA/PAS. In order to obtain the best amount of RE, the adsorption performance of the composite adsorption beads with different RE contents was tested (Figure 4). The results showed that the introduction of an appropriate amount of RE could significantly improve the adsorption capacity for MB and Pb(II) of the adsorption beads, and the adsorption performance was the best when the RE content was 0.6%. The main reason was that the introduction of a moderate amount of RE could improve the network structure, reducing the entanglement of the internal polymer chains, and then release more functional groups that could be complexed with the adsorbent. The ion exchange of RE and the adsorption of functional groups on the polymer achieved synergistic adsorption so as to increase the adsorption capacity. In addition, the introduction of a moderate amount of RE could improve the pore structure of the adsorption beads, narrow the pore size distribution, and increase the mesoporous degree, which was conducive to improving the diffusion of adsorbates into the adsorption beads, and thereby improving the adsorption capability of the adsorbent. As the SA/PASP/RE0.6 adsorption beads had the best adsorption capacity, they were selected as the best adsorbent for conducting the subsequent adsorption experiments.

#### 3.2.2. Influence of External pH on Adsorption

**Figure 5:**
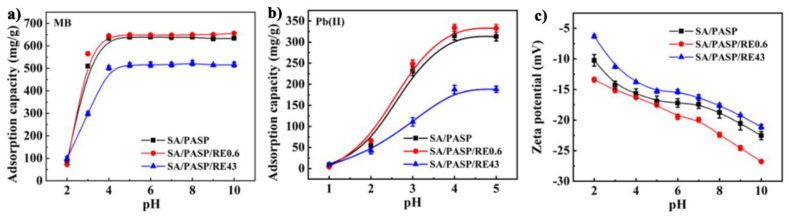
The effect of pH on the adsorption of SA/PASP, SA/PASP/RE0.6, and SA/PASP/RE43 adsorbents toward (**a**) MB and (**b**) Pb(II); and (**c**) the dependence of the Zeta potential of SA/PASP, SA/PASP/RE0.6, and SA/PASP/RE43 on external pH value.

As shown in Figure 5a,b, the adsorption capacity for MB and Pb(II) on the SA/PASP, SA/PASP/RE0.6, and SA/PASP/RE43 beads reached the best values at pH ≥ 4, respectively. By contrast, the adsorption performance of SA/PASP/RE0.6 was the best under each pH condition, indicating that the introduction of a small amount of RE was beneficial to improving the adsorption performance of the composite beads under various pH conditions, which was related to the more negative Zeta potential of SA/PASP/RE0.6 than of SA/PASP (Figure 5c) [52]. The composite beads showed very high adsorption capacity for MB in the pH range of 4–10 and had very good pH resistance. As the beads were ionic adsorbents, large amounts of carboxyl (-COO-) and hydroxyl (-OH) groups were present in their network structure. These groups were sensitive to the change in external pH. When pH < 4, the -COO- group on the beads could protonate to transform into the -COOH group, which reduced the charge density on the adsorbent surface and weakened the complexation ability of functional groups toward MB and Pb(II), resulting in a decrease in the adsorption capacity at pH < 4 [53]. When pH ≥ 4, the part of the -COOH group on the surface of the adsorbent was deprotonated and transformed into the -COO- group, with stronger electrostatic attraction and complexation ability toward MB and Pb(II). At this time, the adsorption capacity was also significantly increased with the increase in pH. When pH > 5.2, Pb(II) ions would precipitate in the form of Pb(OH)_2_ and interfere with the final adsorption results, so pH 5 was selected as the best pH value for evaluating the adsorption of Pb(II) ions on the beads.

#### 3.2.3. Adsorption Isotherms and Kinetics

**Figure 6:**
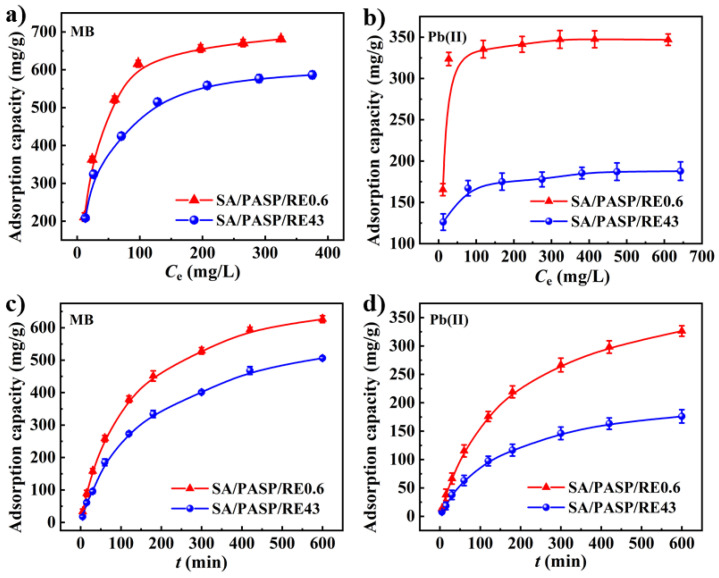
The adsorption isotherm curves of SA/PASP/RE0.6 and SA/PASP/RE43 for the adsorption of (**a**) MB and (**b**) Pb(II) at different equilibrium concentrations; and the effect of contact time on the adsorption of SA/PASP/RE0.6 and SA/PASP/RE43 for (**c**) MB and (**d**) Pb(II).

Figure 6a,b displays the adsorption behaviors of the SA/PASP/RE0.6 and SA/PASP/RE43 composite beads toward MB and Pb(II) at different original concentrations (MB solution: 100–800 mg/L; Pb(II) solution: 50–700 mg/L). The adsorption capacity of the composite beads for MB or Pb(II) increased rapidly at first, and then tended to be flat. The adsorption behavior of MB or Pb(II) on the beads was studied using Freundlich and Langmuir models [54,55]. The fitting results with the Langmuir model showed that the plots of *C*_e_/*q*_e_ versus *C*_e_ for the adsorption of both MB and Pb(II) were straight lines with good linear relationship coefficients (Figures S4a and S5a in Supplementary Materials). In comparison, the *R*^2^ values obtained by fitting with the Freundlich model were significantly lower than those obtained by fitting with the Langmuir model (Table S1 in Supplementary Material). This indicated that the Langmuir model could express the adsorption behavior of MB or Pb(II) on the beads better than the Freundlich model, and that the adsorption process of dye or Pb(II) on the beads was characterized by single-layer adsorption. In addition, in order to make the simulation more accurate, we used a three-parameter Sips model with higher fitting accuracy and greater flexibility to fit the adsorption behavior of MB and Pb(II) on the beads. As shown in Figure S6, the fitting correlation coefficients (*R*^2^) of the composite beads for MB and Pb(II) were greater than 0.99 (Table S2 in Supplementary Material), the heterogeneity factor βα was close to 1, and the isotherm was close to the Langmuir adsorption isotherm model, which further verified that the adsorption behavior of the composite beads toward MB and Pb(II) was in line with the Langmuir adsorption isothermal model, indicating that it was a monolayer adsorption process. By comparing the adsorption capacity of the SA/PASP/RE0.6 and SA/PASP/RE43 beads with that of others, it was found that inexpensive SA/PASP/RE43 with a high content of cheap RE still had a satisfactory adsorption capacity for MB and Pb(II) ions (Table 1). The adsorption capacity for MB and Pb(II) on the beads was higher than that of most other adsorbents. The results demonstrated that the incorporation of a high dosage of clay into the natural polymer network was a universal method to fabricate a low cost, high-performance, and environmentally friendly adsorption material with great application prospects.

Figure 6c,d shows the adsorption kinetic curves of the SA/PASP/RE0.6 and SA/PASP/RE43 beads for MB and Pb(II). In the initial stage (within 100 min), the adsorption process was relatively fast. With extension of the adsorption time, the adsorption sites were continuously occupied and the adsorption rate slowed down significantly, until adsorption saturation was reached. The experimental data were fitted with the pseudo-second-order kinetic model and the pseudo-first-order kinetic model to explore the kinetic adsorption behavior of the composite beads toward MB and Pb(II) ions.

The fitting results with the two kinetic models are shown in Table S3 (Supplementary Material). The fitting results with the pseudo-second-order kinetic model had higher *R*^2^ values than those fitted with the pseudo-first-order kinetic model, and the plots of *t*/*q*_t_ versus *t* were straight lines (Figures S7 and S8 in Supplementary Material). This confirmed that the adsorption of MB and Pb(II) on the SA/PASP/RE0.6 and SA/PASP/RE43 beads was more consistent with the pseudo-second-order kinetic model and revealed that the adsorption of MB and Pb(II) by SA/PASP/RE0.6 and SA/PASP/RE43 was mainly controlled by chemisorption. When the RE content was 43%, the adsorption of MB and Pb(II) by the SA/PASP/RE43 beads conformed to the pseudo-first-order and pseudo-second-order kinetic models (the *R*^2^ values obtained by fitting were very close), indicating that both physical adsorption and chemical adsorption played important roles in the adsorption process. Since RE can adsorb dyes and heavy metals through ion exchange and electrostatic interaction [63], the physically filled RE in the SA/PASP/RE43 beads could adsorb MB and Pb(II) through ion exchange or electrostatic interaction during the adsorption process. The adsorption of dye molecules and heavy metal ions by the carboxyl groups in SA/PASP/RE43 was mainly driven by complexation action (a chemical adsorption process). With continuation of the adsorption process, more and more MB or Pb(II) diffused into the interior network of the beads so that the adsorption capacity gradually increased. At this time, RE in the network structure could generate ion exchange and electrostatic interaction with MB or Pb(II), resulting in a physical adsorption process. Therefore, SA/PASP/RE43 showed a better adsorption capacity even when the content of RE in the beads reached 43%.

#### 3.2.4. Adsorption of MB and Pb(II) in Natural Water

**Figure 7:**
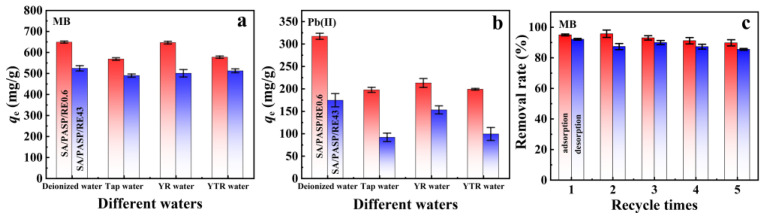
Adsorption capacity of SA/PASP/RE0.6 and SA/PASP/RE43 for (**a**) MB and (**b**) Pb(II); (**c**) Curves of removal rate versus recycle time for expressing reusability.

As the composition of natural water is more complex than that of pure water, the adsorption performance of the composite beads for MB and Pb(II) in natural water (tap water, Yellow River, and Yangtze River) was studied, and the results are shown in Figure 7a,b. The adsorption capacity of the SA/PASP/RE0.6 beads for MB and Pb(II) in Yellow River water was high (adsorption capacity: 643 mg/g for MB, and 211.78 mg/g for Pb(II)), which was close to the adsorption capacity in deionized water. The adsorption capacities of the beads were 567.10 mg/g and 577.80 mg/g for MB and 195.69 mg/g and 197.13 mg/g for Pb(II) in tap water and Yangtze River water, respectively. Similarly, the adsorption capacities of the SA/PASP/RE43 beads for MB and Pb(II) in Yellow River water were 509.5 mg/g and 152.8 mg/g, respectively. The adsorption capacities for MB and Pb(II) in tap water were 487.59 mg/g and 91.95 mg/g, respectively. The adsorption capacities for MB and Pb(II) in Yangtze River water were 512.81 mg/g and 98.56 mg/g, respectively. The main reason for the decrease in MB and Pb(II) adsorption in tap water and Yangtze River water was competition with Mg^2+^, Ca^2+^, and other metal ions on the adsorption sites. The SA/PASP/RE0.6 and SA/PASP/RE43 composite beads with different RE contents showed an excellent adsorption effect for MB and Pb(II) in natural water samples, showing their great application potential in water treatment [64,65].

#### 3.2.5. Reusability

It is important to explore the reusability of composite beads for evaluating their application potential. As shown in Figure 7c, the adsorption rate and desorption rate of the SA/PASP/RE0.6 beads decreased slightly with the increase in the number of cycles. Compared with the first cycle, the recovery rate of the regenerated adsorbent decreased slightly by 7%. After 5 cycles of desorption and regeneration, the adsorption rate of MB on the SA/PASP/RE0.6 composite beads could still reach 85.6%, which further indicated that the SA/PASP/RE0.6 composite beads had good stability and could be recycled and reused for multiple cycles [66].

### 3.3. Adsorption Mechanism

**Figure 8:**
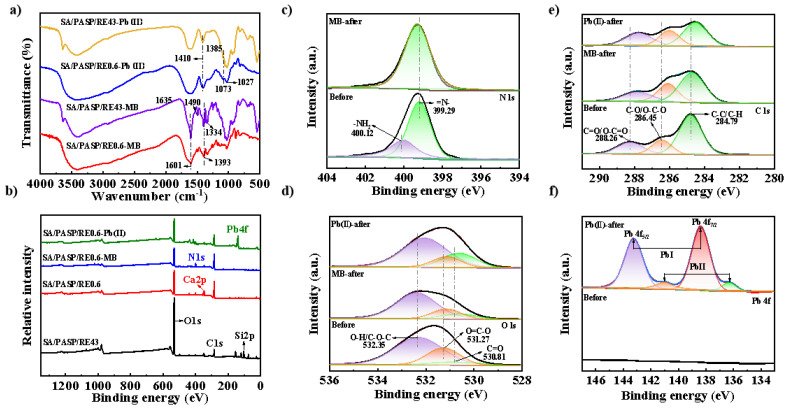
(**a**) FT-IR spectra of SA/PASP/RE0.6-MB, SA/PASP/RE43-MB, SA/PASP/RE0.6-Pb(II), and SA/PASP/RE43-Pb(II); (**b**) survey-scanning XPS spectra of SA/PASP/RE0.6, SA/PASP/RE43, SA/PASP/RE0.6-MB, and SA/PASP/RE43-Pb(II); and fine-scanning XPS spectra of (**c**) N 1s, (**d**) Pb 4f, (**e**) C 1s, and (**f**) O 1s of the SA/PASP/RE0.6 beads before and after adsorption.

The interaction of the SA/PASP/RE0.6 and SA/PASP/RE43 adsorption beads with cationic dye and Pb(II) was studied by FT-IR and XPS analyses (Figure 8). The absorption bands of the functional groups of the SA/PASP/RE0.6 and SA/PASP/RE43 beads decreased or shifted after adsorption of MB due to the interaction between the surface groups of the composite beads and MB molecules [67]. The FT-IR spectra of MB-adsorbed beads (SA/PASP/RE0.6-MB and SA/PASP/RE43-MB) showed that the characteristic absorption peaks of MB appeared at 1601 cm^−1^ (stretching vibration of aromatic C=C and C=N), 1410 cm^−1^ (C-N stretching), 1334 cm^−1^ (overlap of asymmetric stretching within C=N and -C-N rings), and 1083 cm^−1^ (C-H bending vibration). The results showed that MB was adsorbed on the composite beads [68,69]. After adsorption of MB dye, the characteristic peaks of SA/PASP/RE0.6 and SA/PASP/RE43 at 1623 and 1415 cm^−1^ weakened and shifted to 1604 and 1393 cm^−1^, indicating that a strong interaction was present between the carboxylic groups and the dye molecules. After the adsorption of Pb(II) (Figure 8a), the -COO- group in the adsorption beads would combine with Pb(II) to form the Pb…-COO- complex, while the negatively charged -COO- group attracted Pb(II) through electrostatic interaction, and the two actions promoted the adsorption of Pb(II) on the beads.

The N atoms in the SA/PASP/RE0.6 composite beads existed in two chemical states (Figure 8b): the free amino groups (–NH_2_) (BE = 400.12 eV) and the amino =N– structure (BE = 399.29 eV) [70,71]. After adsorption of MB, a slight shift in the N 1s signal peak of the –NH_2_ group toward higher binding energies was observed (Figure 8b), indicating that the N atom in the dye acted as an electron donor during the adsorption process. After adsorption of MB, the N 1s peak of =N- shifted slightly from 400.12 eV to 400.29 eV. The N 1s peak of NH_2_ disappeared. This showed that the -NH_2_ group strongly bound to MB through N-H…N=, NH…O=C, or NH…O-C=O bonding, resulting in complete coverage of the adsorption site by dye molecules. After adsorption of Pb(II), a new peak appeared at 413.5 eV due to the binding of Pb(II) with NH-, and the signal peak of -NH_2_ also shifted to 399.67 eV, indicating that Pb(II) formed a complex with the N-containing group on the PASP chain (Figure S9).

The XPS spectra showed four different peak areas of Pb(II) (Figure 8f). Two main peaks were observed at 143.24 and 138.37 eV, which were attributed to Pb 4f5/2 and Pb 4f7/2, with a relative area ratio of approximately 4:3 (Figure 8f). Although a weak satellite peak of Pb(II) was found at 136.33 eV, the proportion of sub-peaks with low BE values accounted for a large proportion of the Pb 4f peak. The deconvoluted Pb 4f spectrum showed that two component peaks with BE values of 143.24 and 138.37 eV and 136.33 and 141.03 eV could be assigned to Pb^4+^ and Pb^2+^, respectively [72,73]. The C 1s signal peak of the SA/PASP/RE0.6 beads could be divided into three peaks at 288.26 eV, 286.45 eV, and 284.79 eV, which corresponded to the signal peaks of C=O/O-C=O, C-O/O-C-O and C-C/C-H bonds in the beads, respectively [74,75]. After adsorption of MB, the signal peaks at 288.26 eV and 286.45 eV shifted to the low binding energy region (Figure 8c), indicating that the functional groups (i.e., carboxyl, hydroxyl groups) on the beads generated an interaction with the dye molecules.

The continuous transport of MB or Pb(II) from the outer layer to the inner layer enabled the beads to continuously adsorb MB or Pb(II) until saturation adsorption was reached. The O 1s signal peaks of the composite beads could be divided into three peaks at 530.81 eV (C=O groups of PASP), 531.27 eV (carboxylic group of SA and PASP), and 532.30 eV (alcohol hydroxyl group of SA). As shown in Figure 8e, after adsorption of MB and Pb(II), the O 1s peak of the beads shifted to the low binding energy region, indicating that an interaction was present between the oxygen-containing functional groups on the beads and dye or Pb(II) [76].

## 4. Conclusions

The eco-friendly and recyclable composite beads with a network structure and superior adsorption capability were prepared from all-natural sources of SA, PASP, and nanoclay rectorite (RE) in the absence of any hazardous chemicals. The network structure was rich in a large number of functional groups, and so showed good adsorption properties for MB and Pb(II). A small amount of clay in the SA/PASP/RE0.6 adsorbent contributed to the increase in adsorption capacity (MB adsorption capacity: 680.83 mg/g, Pb(II) adsorption capacity: 347.50 mg/g). The addition of 43% clay kept better adsorption capacities of 586.46 mg/g for MB and 187.78 mg/g for Pb(II). Pleasingly, the beads showed excellent capture ability for MB and Pb(II) in actual natural water samples. The adsorption of MB and Pb(II) by the beads was mainly due to the synergistic effect of electrostatic attraction, chemical complexation, pore size adsorption, and ion exchange. Finally, the environmentally friendly super adsorbent beads with high clay content, low swelling rate, and strong adsorption capacity could be prepared by a green and simple process and can be widely used as a cost-effective, efficient, and safe adsorbent for the adsorption and removal of dyes and heavy metals in wastewater.

## Figures and Tables

**Table 1:** Comparison of adsorption capacity (AC) of the all-natural beads with other natural polymer-based adsorbents for MB and Pb(II).

Adsorbates	Adsorbents	Initial Concentration (mg/L)	AC (mg/g)	Ref.
MB	MXene/SA gel beads	160	91.12	[56]
	SA–flax seed ash beads	120	333.0	[57]
	Tannic acid–poly(vinyl alcohol)/SA Beads	160	147.0	[58]
	Magnetic SA/rice husk bio-composite	500	274.9	[59]
	SA/PASP/RE0.6	700	680.83	This work
	SA/PASP/RE43	700	586.46	This work
Pb(II)	Porous cellulose nanofiber–SA beads	800	318	[60]
	Nanochitosan/SA beads	150	178.57	[61]
	Nanocellulose/SA/carboxymethyl-chitosan beads	300	472.59	[62]
	SA/PASP/RE0.6	700	347.50	This work
	SA/PASP/RE43	700	187.78	This work

## Data Availability

The data presented in this study are available upon request from the corresponding author.

## Supplementary Materials

The following supporting information can be downloaded at: https://www.mdpi.com/article/10.3390/nano14090766/s1. Figure S1: The structures of SA, PASP, and RE. Figure S2: (a) SEM images, EDS curves, and corresponding element mapping of C, N, Mg, Al, Ca, and Si elements in SA/PASP/RE0.6; and (b) SEM image, EDS curves, and corresponding element mapping of C, N, Mg, Al, Ca, and Si elements in SA/PASP/RE43. Figure S3: The digital photos of the SA/PASP/RE0.6 composite beads: (a) dry state and (c) wet state; and the digital photos of the SA/PASP/RE43 composite beads: (b) dry state and (d) wet state. (e) A scheme for the ionic crosslinking structure. Figure S4: The linear fitting curves with the Langmuir model (a) and the Freundlich model (b) for the adsorption of MB onto SA/PASP/RE0.6 and SA/PASP/RE43 beads. Figure S5: The linear fitting curves with the Langmuir model (a) and the Freundlich model (b) for the adsorption of Pb(II) onto SA/PASP/RE0.6 and SA/PASP/RE43 beads. Figure S6: The nonlinear fitting curves with the Sips model for the adsorption of MB (a) and Pb(II) (b) onto the SA/PASP/RE0.6 and SA/PASP/RE43 beads. Figure S7: The linear fitting curves with pseudo-second-order (a) and pseudo-first-order (b) kinetic models for the adsorption of MB onto the SA/PASP/RE0.6 and SA/PASP/RE43 beads. Figure S8: The linear fitting curves with pseudo-first-order (a) and pseudo-second-order (b) kinetic models for the adsorption of Pb(II) onto the SA/PASP/RE0.6 and SA/PASP/RE43 beads. Figure S9: N 1s spectra before and after adsorption of Pb(II). Table S1: Adsorption isotherm parameters of the adsorption of MB and Pb(II) onto the composite beads. Table S2: Three-parameter adsorption model fitting results for the adsorption of MB and Pb(II) onto the composite beads. Table S3: Adsorption kinetic parameters for the adsorption of MB and Pb(II) ions onto the composite beads. References [77,78,79,80] are cited in the Supplementary material.

## References

[1] Zhao J.X., Yuan X.S., Wu X.X., Liu L., Guo H.Y., Xu K.M., Zhang L.P., Du G.B. (2023). Preparation of nanocellulose-based aerogel and its research progress in wastewater treatment. Molecules.

[2] Li R., Li Y., Jia X., Yang J., Miao X., Shao D., Wu J., Song H. (2024). 2D/2D ultrathin polypyrrole heterojunct aerogel with synergistic photocatalytic-photothermal evaporation performance for efficient water purification. Desalination.

[3] Jamali M., Akbari A. (2021). Facile fabrication of magnetic chitosan hydrogel beads and modified by interfacial polymerization method and study of adsorption of cationic/anionic dyes from aqueous solution. J. Environ. Chem. Eng..

[4] Sultan M. (2017). Polyurethane for removal of organic dyes from textile wastewater. Environ. Chem. Lett..

[5] Chauhan M., Sharma B., Kumar R., Chaudhary G., Hassan A., Kumar S. (2019). Green synthesis of CuO nanomaterials and their proficient use for organic waste removal and antimicrobial application. Environ. Res..

[6] Yan S., Song H., Li Y., Yang J., Jia X., Wang S., Yang X. (2022). Integrated reduced graphene oxide/polypyrrole hybrid aerogels for simultaneous photocatalytic decontamination and water evaporation. Appl. Catal. B Environ..

[7] Ponce J., Peña J., Román J., Pastor J.M. (2022). Recyclable photocatalytic composites based on natural hydrogels for dye degradation in wastewaters. Sep. Purif. Technol..

[8] Wang Y.W., Wang W.B. (2023). Multiffunctional materials with controllable super-wettability for oil-water separation and removal of pollutants: Design, emerging applications and challenges. Carbon Neutralization.

[9] Wang J., Yao J., Wang L., Xue Q., Hu Z., Pan B. (2020). Multivariate optimization of the pulse electrochemical oxidation for treating recalcitrant dye wastewater. Sep. Purif. Technol..

[10] Shikha S., Pattanayek S.K. (2022). The surface chemical functionality and diffusion mediated adsorption of organophosphates from their low concentration. Chem. Eng. Res. Des..

[11] Zhao W.T., Zhang H., He Q.D., Han L., Wang T.Y., Guo F., Wang W.B. (2022). Controllable synthesis of porous silicate@carbon heterogeneous composite from Coal Gangue waste as eco-friendly superior scavenger of dyes. J. Clean. Prod..

[12] Yan J.H., Li K.R. (2021). A magnetically recyclable polyampholyte hydrogel adsorbent functionalized with β-cyclodextrin and graphene oxide for cationic/anionic dyes and heavy metal ion wastewater remediation. Sep. Purif. Technol..

[13] Nizam N.U.M., Hanafiah M.M., Mahmoudi E., Mohammad A.W., Oyekanmi A.A. (2022). Effective adsorptive removal of dyes and heavy metal using graphene oxide based Pre-treated with NaOH/H_2_SO_4_ rubber seed shells synthetic graphite Precursor: Equilibrium Isotherm, kinetics and thermodynamic studies. Sep. Purif. Technol..

[14] Kalmykova Y., Strömvall A., Steenari B.-M. (2008). Adsorption of Cd, Cu, Ni, Pb and Zn on Sphagnum peat from solutions with low metal concentrations. J. Hazard. Mater..

[15] Wang X.L., Li Y., Huang J., Zhou Y.Z., Li B.L., Liu D.B. (2019). Efficiency and mechanism of adsorption of low concentration uranium in water by extracellular polymeric substances. J. Environ. Radioactiv..

[16] Ma W.Y., Liu X.Y., Lu H., He Q.D., Ding K., Wang X.H., Wang W.B., Guo F. (2023). Chitosan-based composite hydrogel with a rigid-in-flexible network structure for pH-universal ultra-efficient removal of dye. Int. J. Biol. Macromol..

[17] Guo Y.P., Hasi Q.M., Yu J.L., Guo Y.Y., Song L.Y., Wu S., Luo X.P., Chen L.H. (2023). Carboxymethyl cellulose/sulfonated conjugated microporous polymer composite aerogel for efficient pollution removal and water evaporation. Sep. Purif. Technol..

[18] Wang W.B., Zhao W.T., Zhang H., Xu J., Zong L., Kang Y.R., Wang A.Q. (2021). Mesoporous polymetallic silicate derived from naturally abundant mixed clay: A potential robust adsorbent for removal of cationic dye and antibiotic. Powder Technol..

[19] Lai K.C., Lee L.Y., Hiew B.Y.Z., Thangalazhy-Gopakumar S., Gan S. (2019). Environmental application of three-dimensional graphene materials as adsorbents for dyes and heavy metals: Review on ice-templating method and adsorption mechanisms. J. Environ. Sci..

[20] Wang Y.W., Liu X.Y., He Q.D., Lu H., Guo F., Zhang Y.J., Wang W.B. (2022). One-step efficient separation of heavy/light oils, dyes and water by simple filtration with a 3D architecture of functional mesh and sisal fiber felt. Sep. Purif. Technol..

[21] Hou P., Xing G., Tian L., Zhang G., Wang H., Yu C., Li Y., Wu Z. (2019). Hollow carbon spheres/graphene hybrid aerogels as high-performance adsorbents for organic pollution. Sep. Purif. Technol..

[22] He Q.D., Liu X.Y., Wang Y.W., Ding K., Ge H.W., Xie C.Z., Wang W.B., Guo F. (2022). Circular conversion of waste rectorite@dye to efficient and pH-resistant heterogeneous silicate adsorbents for cyclic and complete dye removal. Appl. Clay Sci..

[23] Wang W.B., Wang A.Q. (2022). Perspectives on green fabrication and sustainable utilization of adsorption materials for wastewater treatment. Chem. Eng. Res. Des..

[24] Li C., Wang X., Meng D., Zhou L. (2018). Facile synthesis of low-cost magnetic biosorbent from peach gum polysaccharide for selective and efficient removal of cationic dyes. Int. J. Biol. Macromol..

[25] Metin A., Dogan D., Can M. (2020). Novel magnetic gel beads based on ionically crosslinked sodium alginate and polyanetholesulfonic acid: Synthesis and application for adsorption of cationic dyes. Mater. Chem. Phys..

[26] Rahman S., Chowdhury D. (2022). Guar gum-sodium alginate nanocomposite film as a smart fluorescence-based humidity sensor: A smart packaging material. Int. J. Biol. Macromol..

[27] Thakur S., Pandey S., Arotiba O.A. (2016). Development of a sodium alginate-based organic/inorganic superabsorbent composite hydrogel for adsorption of methylene blue. Carbohyd. Polym..

[28] Wang X., Zhang H., He Q.D., Xing H.F., Feng K., Guo F., Wang W.B. (2022). Core-shell alginate beads as green reactor to synthesize grafted composite beads to efficiently boost single/co-adsorption of dyes and Pb(II). Int. J. Biol. Macromol..

[29] Shi S., Wu Y., Wang Y., Yu J., Xu Y. (2017). Synthesis and characterization of a biodegradable polyaspartic acid/2-amino-2-methyl-1-propanol graft copolymer and evaluation of its scale and corrosion inhibition performance. RSC Adv..

[30] Ma M.Z., Chen H.Z., Zhang W., Feng E.J., Li X.J., Li F.Q., Xu S.F., Li Y.W. (2022). Novel poly(amino acid)-type superplasticizers with enhanced dispersing performance for Portland cement doped with clay impurities. Colloids Surf. A.

[31] Budai-Szűcs M., Kiss E.L., Szilágyi B.Á., Szilágyi A., Gyarmati B., Berkó S., Kovács A., Horvát G., Aigner Z., Soós J. (2018). Mucoadhesive cyclodextrin-modified thiolated poly(aspartic acid) as a potential ophthalmic drug delivery system. Polymers.

[32] Chai C.X., Xu Y.H., Xu Y., Liu S.H., Zhang L. (2020). Dopamine-modified polyaspartic acid as a green corrosion inhibitor for mild steel in acid solution. Eur. Polym. J..

[33] Ma G.F., Yang Q., Ran F.T., Dong Z.B., Lei Z.Q. (2015). High performance and low cost composite superabsorbent based on polyaspartic acid and palygorskite clay. Appl. Clay Sci..

[34] Bi R., Yin D., Zhang S.Z., Zhang R., Chen F.F. (2022). Efficient removal of Pb(II) and Hg(II) with eco-friendly polyaspartic acid/ layered double hydroxide by host-guest interaction. Appl. Clay Sci..

[35] Liu L., Gao Z., Su X., Chen X., Jiang L., Yao J. (2015). Adsorption removal of dyes from single and binary solutions using a cellulose-based bioadsorbent. ACS Sustain. Chem. Eng..

[36] Xiao W.D., Xiao L.P., Xiao W.Z., Liu K., Zhang Y., Zhang H.Y., Sun R.C. (2022). Cellulose-based bio-adsorbent from TEMPO-oxidized natural loofah for effective removal of Pb(II) and methylene blue. Int. J. Biol. Macromol..

[37] Santagata M., Johnston C.T. (2022). A study of nanoconfined water in halloysite. Appl. Clay Sci..

[38] Wang B.Y., Zhang W.X., Jin X.C., Su G.H. (2021). A diffusion model for the swelling of compacted Na-montmorillonite in water. Appl. Clay Sci..

[39] Uddin M.K. (2017). A review on the adsorption of heavy metals by clay minerals, with special focus on the past decade. Chem. Eng. J..

[40] Ngulube T., Gumbo J.R., Masindi V., Maity A. (2017). An update on synthetic dyes adsorption onto clay based minerals: A state-of-art review. J. Environ. Manag..

[41] Cao X., Chen D., Gu H.Z., Huang A., Ni H.W. (2019). Improved bonding properties of rectorite clay slurry after wet/dry grinding. Appl. Clay Sci..

[42] Zhao Y., Shao Z.Y., Chen C.L., Hu J., Chen H.L. (2014). Effect of environmental conditions on the adsorption behavior of Sr(II) by Na-rectorite. Appl. Clay Sci..

[43] Yang L.L., Ma X.Y., Guo N.N. (2012). Sodium alginate/Na^+^-rectorite composite microspheres: Preparation, characterization, and dye adsorption. Carbohyd. Polym..

[44] Yang L., Ma X., Guo N. (2012). Simultaneous reduction of Cr (VI) and degradation of tetracycline hydrochloride by a novel iron-modified rectorite composite through heterogeneous photo-Fenton processes. Carbohyd. Polym..

[45] Khan S.U., Sultan M., Islam A., Sabir A., Hafeez S., Bibi I., Ahmed M.N., Khan S.M., Khan R.U., Iqbal M. (2021). Sodium alginate blended membrane with polyurethane: Desalination performance and antimicrobial activity evaluation. Int. J. Biol. Macromol..

[46] Oyarce E., Santander P., Butter B., Pizarro G.D., Sanchez J. (2021). Use of sodium alginate biopolymer as an extracting agent of methylene blue in the polymer-enhanced ultrafiltration technique. J. Appl. Polym. Sci..

[47] Zhang B., Zhou D., Lv X., Xu Y., Cui Y. (2013). Synthesis of polyaspartic acid/3-amino-1H-1,2,4-triazole-5-carboxylic acid hydrate graft copolymer and evaluation of its corrosion inhibition and scale inhibition performance. Desalination.

[48] Chen Y., Chen X., Liang Y. (2020). Synthesis of polyaspartic acid/graphene oxide grafted copolymer and evaluation of scale inhibition and dispersion performance. Diam. Relat. Mater..

[49] Feng Z., Liu D., Ma X. (2016). The rectorite/carbon composites: Fabrication, modification and adsorption. Chemosphere.

[50] Shen Y., Yu X., Wang Y. (2019). Facile synthesis of modified rectorite (M-REC) for effective removal of anionic dye from water. J. Mol. Liq..

[51] Sing K.S.W., Everett D.H., Haul R.A.W., Moscou L., Pierotti R.A., Rouquerol J., Siemieniewska T. (1985). Physical and biophysical chemistry division commission on colloid and surface chemistry including catalysis. Pure Appl. Chem..

[52] Zeng L., Xie M., Zhang Q., Kang Y., Guo X., Xiao H., Peng Y., Luo J. (2015). Chitosan/organic rectorite composite for the magnetic uptake of methylene blue and methyl orange. Carbohyd. Polym..

[53] Zheng Y., Wang A. (2009). Evaluation of ammonium removal using a chitosan-g-poly (acrylic acid)/rectorite hydrogel composite. J. Hazard. Mater..

[54] Langmuir I. (1916). The constitution and fundamental properties of solids and liquids. Part I. Solids. J. Am. Chem. Soc..

[55] Freundlich H. (1906). Over the adsorption in solution. J. Phys. Chem..

[56] Zhang Z.H., Xu J.Y., Yang X.L. (2021). MXene/sodium alginate gel beads for adsorption of methylene blue. Mater. Chem. Phys..

[57] Işık B., Uğraşkan V. (2021). Adsorption of methylene blue on sodium alginate-flax seed ash beads: Isotherm, kinetic and thermodynamic studies. Int. J. Biol. Macromol..

[58] Hu T., Liu Q., Gao T., Dong K., Wei G., Yao J. (2018). Facile preparation of tannic acid-poly(vinyl alcohol)/sodium alginate hydrogel beads for methylene blue removal from simulated solution. ACS Omega.

[59] Alver E., Metin A.Ü., Brouers F. (2020). Methylene blue adsorption on magnetic alginate/rice husk bio-composite. Int. J. Biol. Macromol..

[60] Zhao H., Ouyang X.-K., Yang L.-Y. (2021). Adsorption of lead ions from aqueous solutions by porous cellulose nanofiber–sodium alginate hydrogel beads. J. Mol. Liq..

[61] Ablouh E., Essaghraoui A., Eladlani N., Rhazi M., Taourirte M. (2019). Uptake of Pb(II) onto nanochitosan/sodium alginate hybrid beads: Mechanism and kinetics study. Water Environ. Res..

[62] Li W., Zhang L., Hu D., Yang R., Zhang J., Guan Y., Lv F., Gao H. (2021). A mesoporous nanocellulose/sodium alginate/carboxymethyl-chitosan gel beads for efficient adsorption of Cu^2+^ and Pb^2+^. Int. J. Biol. Macromol..

[63] Li Z., Chang P., Jiang W. (2019). Mechanisms of Cu^2+^, triethylenetetramine (TETA), and Cu-TETA sorption on rectorite and its use for metal removal via metal-TETA complexation. J. Hazard. Mater..

[64] Shi J., Guo C., Lei C., Liu Y., Hou X., Zheng X., Hu Q. (2022). High-performance biochar derived from the residue of Chaga mushroom (*Inonotus obliquus*) for pollutants removal. Bioresour. Technol..

[65] Zhou L., Liu J., Lu A., Shen J., Xu J., Jiang H. (2022). Controllable synthesis of cubic magnetic MgFe_2_O_4_ derived from MgFe-LDHs for efficient removal of methyl orange. Chem. Eng. J..

[66] Zhang W., Ou J., Tang M., He Q., Long A., Luo S., Sun S., Wan J., Gao Y., Zhou L. (2022). Physically-crosslinked activated CaCO_3_/polyaniline-polypyrrole-modified GO/alginate hydrogel sorbent with highly efficient removal of copper(II) from aqueous solution. Chem. Eng. J..

[67] Kwak H.W., Hong Y., Lee M.E., Jin H.-J. (2018). Sericin-derived activated carbon-loaded alginate bead: An effective and recyclable natural polymer-based adsorbent for methylene blue removal. Int. J. Biol. Macromol..

[68] Heybet E.N., Ugraskan V., Isik B., Yazici O. (2021). Adsorption of methylene blue dye on sodium alginate/polypyrrole nanotube composites. Int. J. Biol. Macromol..

[69] Gao T., Guan G., Wang X., Lou T. (2022). Electrospun molecularly imprinted sodium alginate/polyethylene oxide nanofibrous membranes for selective adsorption of methylene blue. Int. J. Biol. Macromol..

[70] Xiao J., Lv W., Xie Z., Tan Y., Song Y., Zheng Q. (2016). Environmentally friendly reduced graphene oxide as a broad-spectrum adsorbent for anionic and cationic dyes via π-π interaction. J. Mater. Chem. A.

[71] Feng Y., Wang H., Xu J., Du X., Cheng X., Du Z., Wang H. (2021). Fabrication of MXene/PEI functionalized sodium alginate aerogel and its excellent adsorption behavior for Cr(VI) and Congo Red from aqueous solution. J. Hazard. Mater..

[72] Fiorito E., Porcedda G., Brundu L., Passiu C., Atzei D., Ennas G., Elsener B., Fantauzzi M., Rossia A. (2022). Calcium carbonate as sorbent for lead removal from wastewaters. Chemosphere.

[73] Borup R.L., Sauer D.E., Stuve E.M. (1993). An exsitu study of electrodeposited lead on platinum (111) I. Examination of the surface redox behavior of lead and dynamic emersion. Surf. Sci..

[74] Shi T., Xie Z., Zhu Z., Shi W., Liu Y., Liu M. (2022). Highly efficient and selective adsorption of heavy metal ions by hydrazide-modified sodium alginate. Carbohyd. Polym..

[75] Dong K., Xu K., Wei N., Fang Y., Qin Z. (2022). Three-dimensional porous sodium alginate/gellan gum environmentally friendly aerogel: Preparation, characterization, adsorption, and kinetics studies. Chem. Eng. Res. Des..

[76] Wang W., Fan M., Ni J., Peng W., Cao Y., Li H., Huang Y., Fan G., Zhao Y., Song S. (2022). Efficient dye removal using fixed-bed process based on porous montmorillonite nanosheet/poly(acrylamide-co-acrylic acid)/sodium alginate hydrogel beads. Appl. Clay Sci..

[77] Ho Y.S., McKay G.A. (1998). comparison of chemisorption kinetic models applied to pollutant removal on various sorbents. Process Saf. Environ..

[78] Al-Ghouti M.A., Da’ana D.A. (2020). Guidelines for the use and interpretation of adsorption isotherm models: A review. J. Hazard. Mater..

[79] Fernandes E.P., Silva T.S., Carvalho C.M., Selvasembian R., Chaukura N., Oliveira L.M.T.M., Meneghetti S.M.P., Meili L. (2021). Efficient adsorption of dyes by γ-alumina synthesized from aluminum wastes: Kinetics, isotherms, thermodynamics and toxicity assessment. J. Environ. Chem. Eng..

[80] Deng J.L., Yang L.L., Liang G.Z. (2013). Preparation, characterization and swelling behaviors sodium alginate-graft-acrylic acid/Na^+^ rectorite superabsorbent composites. J. Inorg. Organomet. Polym. Mater..

