# Supplementary material for: Biopolymer Meets Nanoclay: Rational Fabrication of Superb Adsorption Beads from Green Precursors for Efficient Capture of Pb(II) and Dyes
Source: Nanomaterials (Basel). 2024 Apr 26;14(9):766. doi: 10.3390/nano14090766 (PMC11085593; doi:10.3390/nano14090766)
Supplement: Supplementary file 1 [file nanomaterials-14-00766-s001.zip › nanomaterials-2977064-supplementary.pdf]

Supplementary Material

Biopolymer Meets Nanoclay: Rational Fabrication of Superb Adsorption Beads From Green Precursors for Efficient Capture of Pb(II) and Dyes

Jie Qi,† Xue Wang,† Huan Zhang, Xiangyu Liu, Wenbo Wang, Qingdong He, Fang Guo*

College of Chemistry and Chemical Engineering, Inner Mongolia University, Hohhot 010021, China; jieino@163.com (J.Q.); 542446320@qq.com (X. W.); hzhang1989@163.com (H. Z.); lxy13257244737@163.com (X.L.); wangwenbo@imu.edu.cn (W.W.); 18847744604@163.com (Q.H.); guofang@imu.edu.cn (F. G.)

* Correspondence: guofang@imu.edu.cn (F.G.)

†These authors contribute equally to the paper.

Part I: Equations and characterizations

1.1 Equations

The experimental data were fitted with kinetic mathematical models: pseudo-first order kinetic model (Equation S1) and pseudo-second order kinetic models (Equation S2).

$$\frac{t}{q_t} = \frac{1}{k_2 q_e^2} + \frac{t}{q_e}$$

(S1)

$$\mathrm{Log}(q_e - q_t) = \log q_e - \frac{k_1 t}{2.303}$$

(S2)

where q_t and q_e are the adsorption capacity at time t and at adsorption equilibrium, respectively (mg/g), k_1 (min^{-1}) and k_2 (g/(mg·min) are pseudo-first order and pseudo-second order kinetic constants [77].

The adsorption data were fitted with different adsorption isotherm models: Langmuir model (Equation S3) , Freundlich model(Equation S4) and Sips model (Equation S5) [78].

$$q_e = k_f C_e^{\frac{1}{n}} \quad \text{(S3)}$$

$$q_e = \frac{q_m k_L C_e}{1 + C_e k_L} \quad \text{(S4)}$$

$$\mathrm{q_e} = \frac{\mathrm{k_S C_e^{\beta_S}}}{1 + \mathrm{a_S C_e^{\beta_S}}} \quad \text{(S5)}$$

where q_e is the adsorption capacity at adsorption equilibrium state (mg/g), q_m is the maximum adsorption capacity (mg/g); K_L is the Langmuir constant related to theoretical monolayer adsorption (L/mg); C_e is the concentration of adsorbate in solution at adsorption equilibrium state (mg/L); K_f is the Freundlich adsorption isotherm constant [(mg/L)(L/g) 1/n]; n is a constant related to the intensity of adsorption [79]. K_S is the Sips model isotherm constant (L/g); a_S the Sips model constant (L/mg) and β_S the Sips model exponent.

1.2 Characterizations

The surface morphologies of composite beads were examined using a Field Emission Scanning Electron microscope (SUPRA55, Carl Zeiss, Germany). The microscopic structure was observed with a high resolution transmission electron microscope (JEM-2100f, JEOL, Japan). Fourier transform infrared (FTIR) spectra were measured with a Nicolet iS10 spectrophotometer (Thermo Fisher, USA). The crystallization state of the composite beads with different clay content was analyzed by a X-ray powder diffractometer (D8 Advance, Bruker, Germany) equipped with a Cu-Kα radiation source (40 kV, 40 mA). X-ray photoelectron spectroscopy (XPS) was measured with an ESCALAB Xi+ spectrophotometer (Thermo Fisher Scientific, American). After adsorption process, the composite beads were removed from the solution, and the concentration of residual dye in the solution was tested with a UV1900i UV–Vis spectrophotometer (Shimadzu, Japan). Zeta potentials were tested with a NanoBrook 90Plus Zeta Potentiometer (Brookhaven, USA). The concentration of Pb(II) ions was measured with a ZEEnit 700P Atomic Absorption Spectrometer (Analytic Jena, Germany). The content of Na in SA was determined with an Avio 200 ICP instrument (Perking Elmer, USA).

Part II. Supplementary Figures

SA

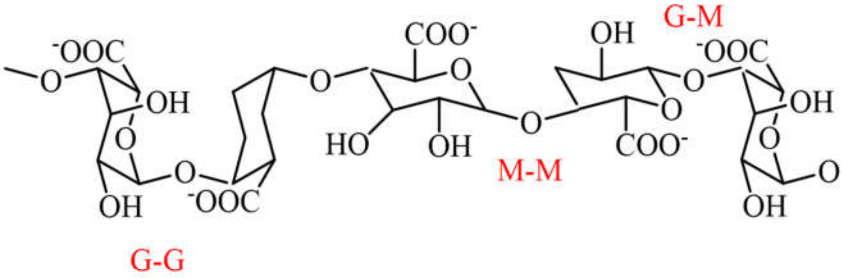

PASP

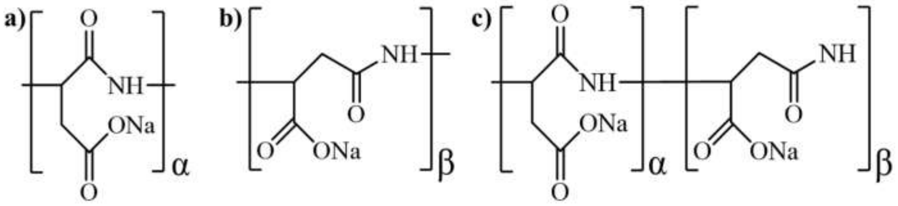

RE

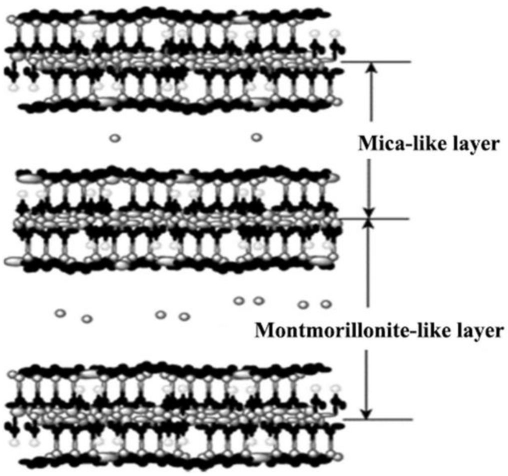

Figure S1. The structure scheme of SA, PASP, and RE [80].

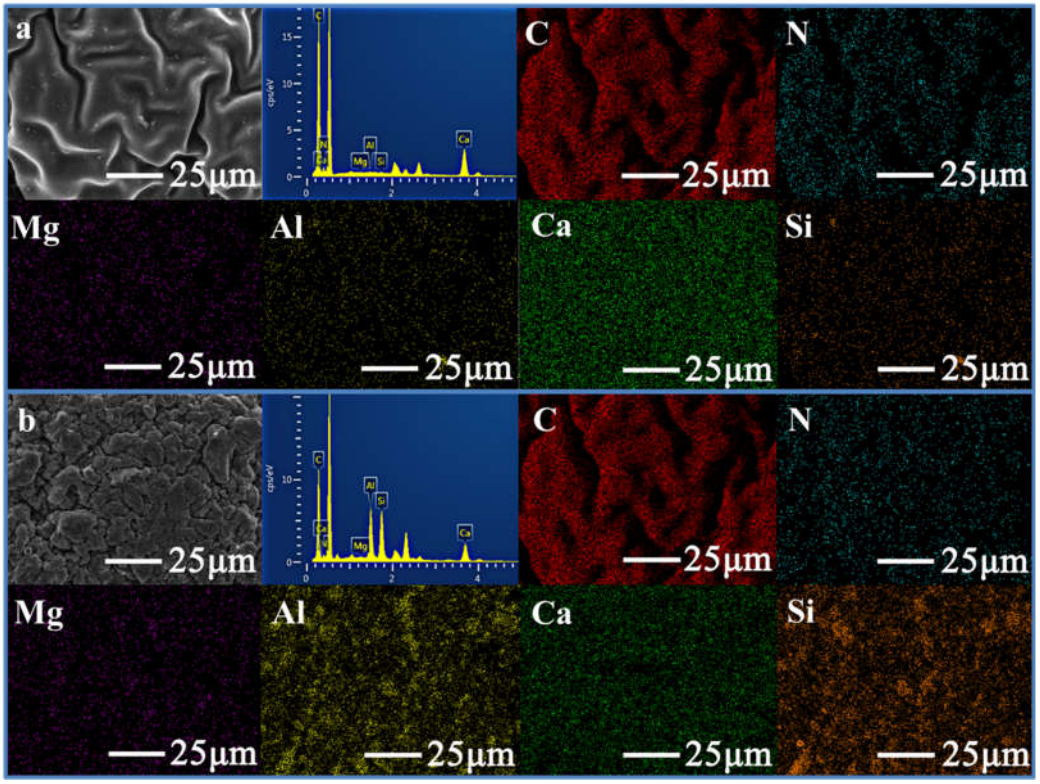

Figure S2. (a) SEM images, EDS curves and corresponding element mapping of C, N, Mg, Al, Ca, Si elements in SA/PASP/RE0.6; and (b) SEM image, EDS curves and corresponding element mapping of C, N, Mg, Al, Ca, Si elements in SA/PASP/RE43.

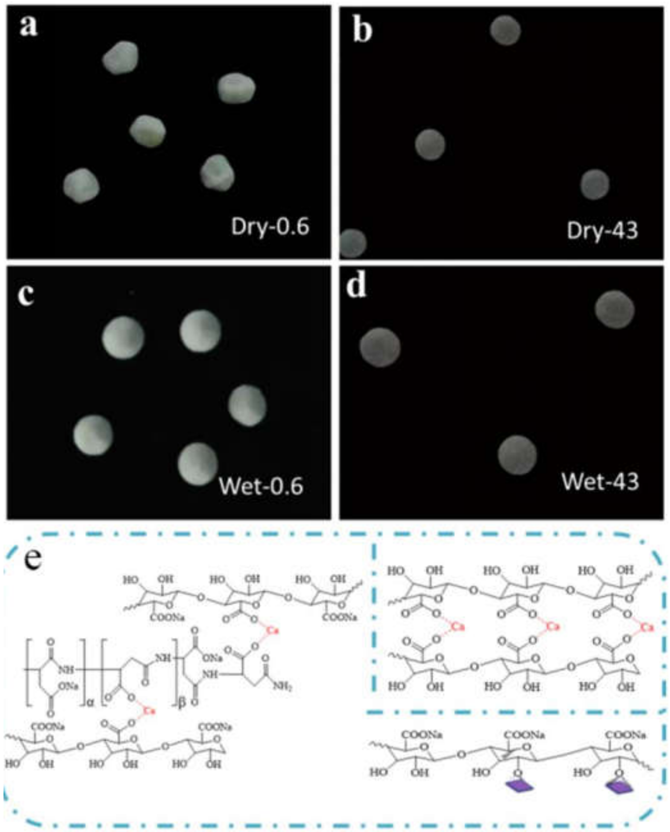

Figure S3. The digital photos of the SA/PASP/RE0.6 composite beads: (a) Dry state and (c) Wet-state; and the digital photos of the SA/PASP/RE43 composite beads: (b) Dry and (d) Wet-state; and (e) a scheme for the ion-crosslinking structure.

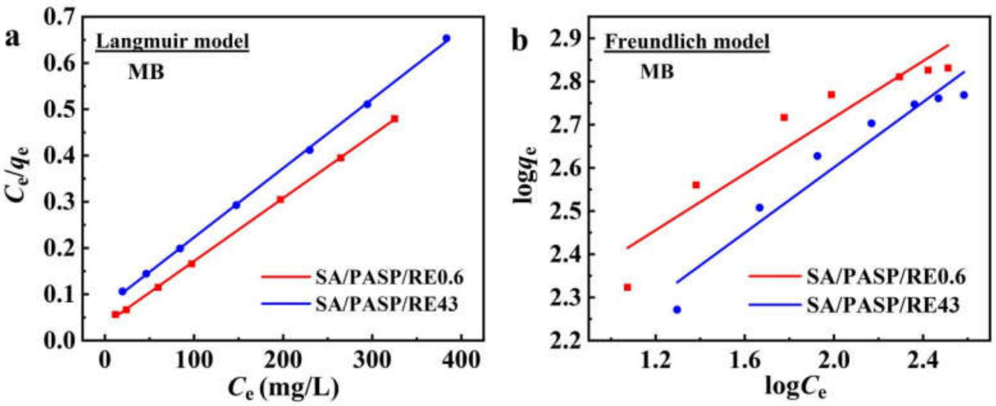

Figure S4. The linear fitting curves with Langmuir model (a) and the Freundlich model (b) for the adsorption of MB onto SA/PASP/RE0.6 and SA/PASP/RE43 beads.

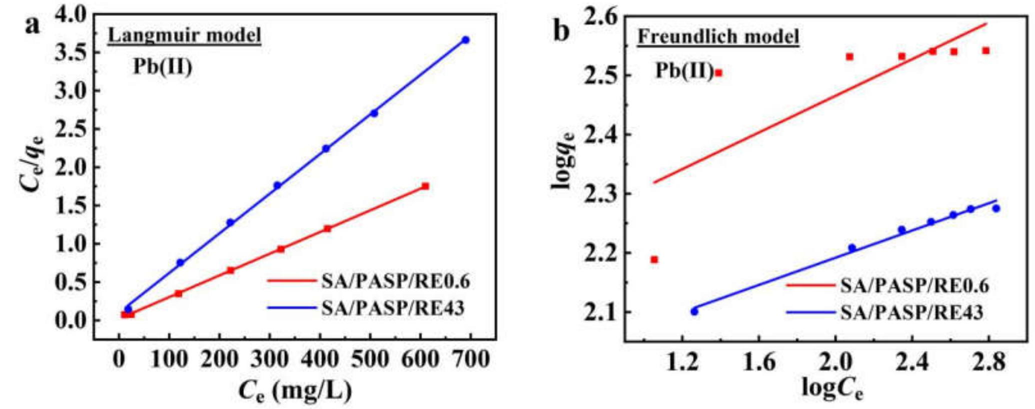

Figure S5. The linear fitting curves with Langmuir model (a) and the Freundlich model (b) for the adsorption of Pb(II) onto SA/PASP/RE0.6 and SA/PASP/RE43 beads.

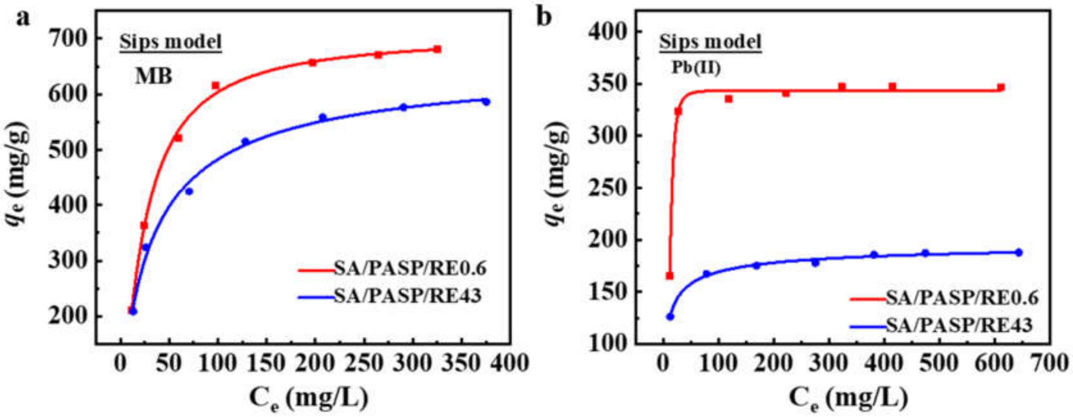

Figure S6. The nonlinear fitting curves with Sips model for the adsorption of MB (a) and Pb(II) (b) onto SA/PASP/RE0.6 and SA/PASP/RE43 beads.

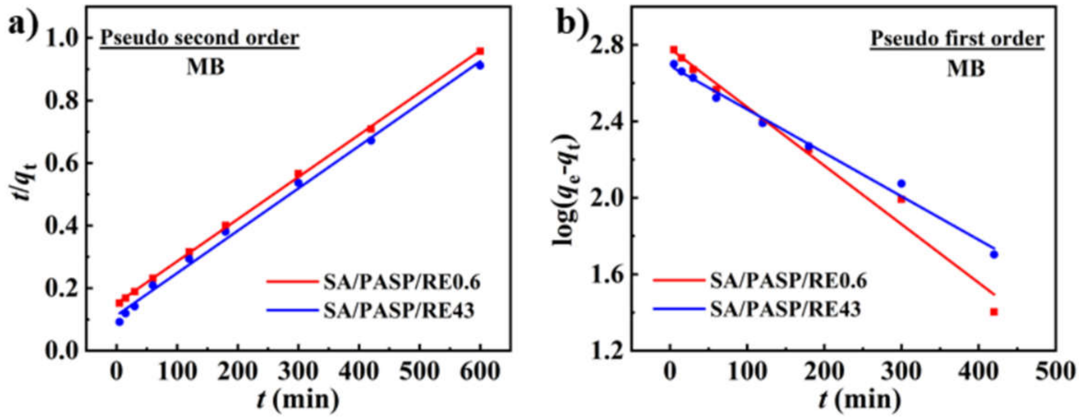

Figure S7. The linear fitting curves with pseudo-second order (a) and pseudo-first order (b) kinetic models for the adsorption of MB onto the SA/PASP/RE0.6 and SA/PASP/RE43 beads.

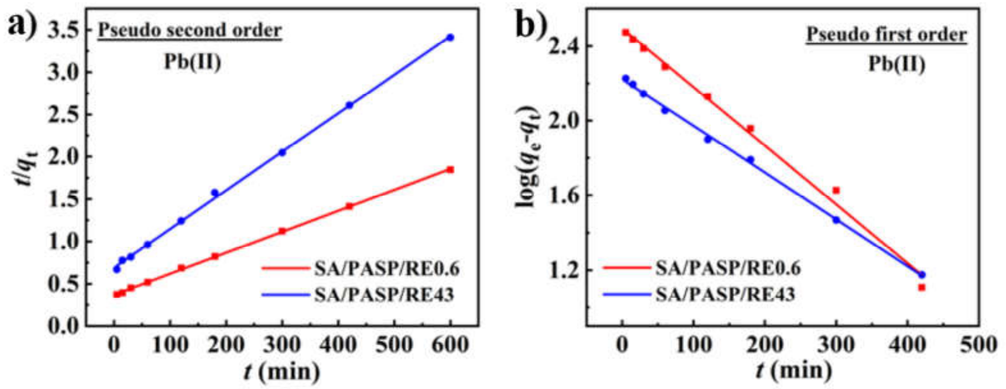

Figure S8. The linear fitting curves with pseudo-first order (a) and pseudo-second order (b) kinetic models for the adsorption of Pb(II) onto the SA/PASP/RE0.6 and SA/PASP/RE43 beads.

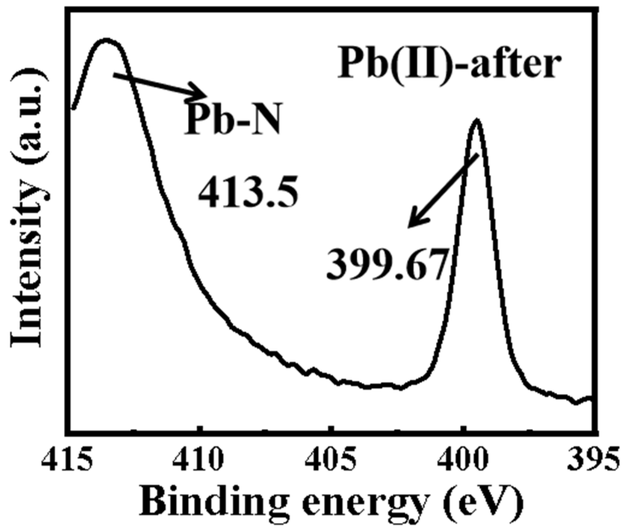

Figure S9. N1s spectra before and after adsorption of Pb(II).

Part III. Supplementary Tables

Table S1. Two-parameter adsorption model of the adsorption of MB and Pb(II) onto the composite beads.

Adsorbates	Samples	Langmuir			Freundlich		
		K_L(L/mg)	Q_m (mg/g)	R^2	K_F (L/mg)	n	R^2
MB	SA/PASP/RE0.6	0.035	735.29	0.9997	2.064	3.06	0.8942
	SA/PASP/RE43	0.073	666.67	0.9995	1.844	1.28	0.9362
Pb(II)	SA/PASP/RE0.6	0.021	353.36	0.9996	2.156	6.46	0.5243
	SA/PASP/RE43	0.102	193.05	0.9992	1.961	8.69	0.9826

Table S2. Three-parameter adsorption model of the adsorption of MB and Pb(II) onto the composite beads.

Adsorbates	Samples	Sips			
		Ks(L/g)	βs	as(L/mg)	R2
MB	SA/PASP/RE0.6	14.8425	1.2211	0.0209	0.9977
	SA/PASP/RE43	36.1505	0.8465	0.0545	0.9943
Pb(II)	SA/PASP/RE0.6	0.1151	1.3266	0.0003	0.9959
	SA/PASP/RE43	85.955	0.8533	0.4296	0.9932

Table S3. Adsorption kinetic parameters for the adsorption of MB and Pb(II) ions onto the composite beads.

Adsorbates	Samples	Pseudo-first-order model			Pseudo-second order model		
		$K_1 \times 10^2$ (min^{-1})	$q_{e,cal,1}$ (mg/g)	R^2	$K_2 \times 10^5$ (min^{-1})	$q_{e,cal,2}$ (mg/g)	R^2
MB	SA/PASP/RE0.6	0.0070	602.56	0.9790	1.24	729.93	0.9995
	SA/PASP/RE45	0.0052	489.33	0.9921	0.99	636.94	0.9965
Pb(II)	SA/PASP/RE0.6	0.0073	309.03	0.9926	1.64	404.86	0.9996
	SA/PASP/RE45	0.0058	165.96	0.9984	3.01	219.30	0.9991

References

[77] Ho, Y.S.; McKay, G. A comparison of chemisorption kinetic models applied to pollutant removal on various sorbents. *Process Saf. Environ.* **1998**, *76*, 332-340.

[78] Al-Ghouti, M.A.; Da'ana, D.A. Guidelines for the use and interpretation of adsorption isotherm models: A review. *J. Hazard. Mater.* **2020**, *393*, 122383.

[79] Fernandes, E.P.; Silva, T.S.; Carvalho, C.M.; Selvasembian, R.; Chaukura, N.; Oliveira, L.M.T.M.; Meneghetti, S.M.P.; Meili, L. Efficient adsorption of dyes by γ-alumina synthesized from aluminum wastes: Kinetics, isotherms, thermodynamics and toxicity assessment. *J. Environ. Chem. Eng.* **2021**, *9*, 106198.

[80] Deng, J.L.; Yang, L.L.; Liang, G.Z. Preparation, characterization and swelling behaviors sodium alginate-graft-acrylic acid/Na^+ rectorite superabsorbent composites. *J. Inorg. Organomet. Polym. Mater.* **2013**, *23*, 525-532.
